# Axillary nerve position in the anterosuperior approach of the shoulder: a cadaveric study

**DOI:** 10.1590/1413-78522015230100960

**Published:** 2015

**Authors:** Roberto Yukio Ikemoto, Luis Gustavo Prata Nascimento, Rogerio Serpone Bueno, Luiz Henrique Oliveira Almeida, Eric Strose, Joel Murachovsky

**Affiliations:** Faculdade de Medicina do ABC, Santo André, SP, Brazil, Faculdade de Medicina do ABC, Santo André, SP, Brazil

**Keywords:** Shoulder/surgery, Anatomy, regional, Humerus, Cadaver

## Abstract

**Objective:**

To determine the distance between the axillary nerve and the antero-lateral (AL) edge of the acromion, its anatomical variability and relationship to humeral length and body height.

**Methods:**

Twenty-two shoulders were dissected. The anterosuperior (AS) approach was used; the deltoid was detached from the acromion and the distance between the AL portion and the axillary nerve was measured and submitted to statistical analysis.

**Results:**

The distance varied from 4.3 to 6.4 cm (average 5.32 ± 0.60 cm). The axillary nerve distance increased as the humeral size (p<0.05) and the height of each cadaver increased. However, the correlation with the specimens height was not significant (p=0.24).

**Conclusions:**

The distance between the acromion and the axillary nerve on the AS approach was 5.32 ± 0.60 cm in both shoulders, and increasing the humeral length there is also an increase in the axillary nerve distance. Level of Evidence IV, Case Series - Anatomic Study.

## INTRODUCTION

The antero-superior (AS) approach of the shoulder described by Makenzie has also been used by Copeland to "resurfacing" arthroplasty and by other authors to reverse arthroplasty and open reduction with internal fixation of fractures.^1^^-^ 4 Its main advantage is the quick approach to the proximal portion of the humerus through the deltoid, among its anterior and medial portions. Unlike the deltopectoral approach, which can be extended distally, the antero-superior approach has its distal extent limited by the axillary nerve that crosses through by about five centimeters from the lateral edge of the acromion.^5^^,^ 6 Despite the nerve location and its anatomic relationships are well described in the literature regarding the deltopectoral approach, 5 ^,^ 7 ^-^ 14 its relationship to the anterolateral edge of the acromion in the anterolateral approach has not been described. The exact nerve location is important to prevent injuries that may damage the shoulder function.

The objective of this study is to determine the distance between the axillary nerve and the anterolateral border of the acromion, besides analyzing their anatomical variability, its relation to the length of the humerus and to body height.

## MATERIALS AND METHODS

Between March and September 2007, 22 shoulders (11 cadavers) were dissected. Nine specimens were male and two were female. The mean age was 45±12 years old (ranging from 25 to 55 years old) and the mean height was 1.63±0.08 m (range 1.52 to 1.82 m).

The sample was calculated so that there was a 95% confidence interval for the variables under study.

**Figure 1.**
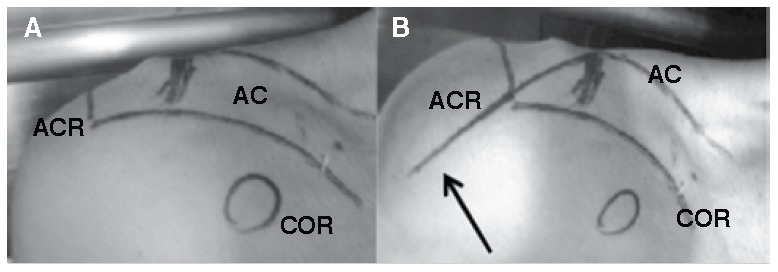
(A) Reference points of the antero-superior access: antero-lateral edge of the acromion (Ac), acromioclavicular joint (AC) and coracoid process (Cor); (B) Incision mark on skin (black arrow).

The specimens were dissected with the shoulder positioned in neutral rotation by an assistant who kept the elbow at a 90° flexion and arm next to the body while palpating both epicondyle of the elbow. Before skin incision, the following parameters were identified: coracoid process, anterolateral border of the acromion, spine of the scapula and the acromioclavicular joint (AC). (Figure 1A-B) The incision was made starting from the AC joint across the front of the acromion and extended distally through the muscle belly of the deltoid, 5-7 cm from the anterolateral edge of the acromion. (Figure 1A-B)

**Figure 2.**
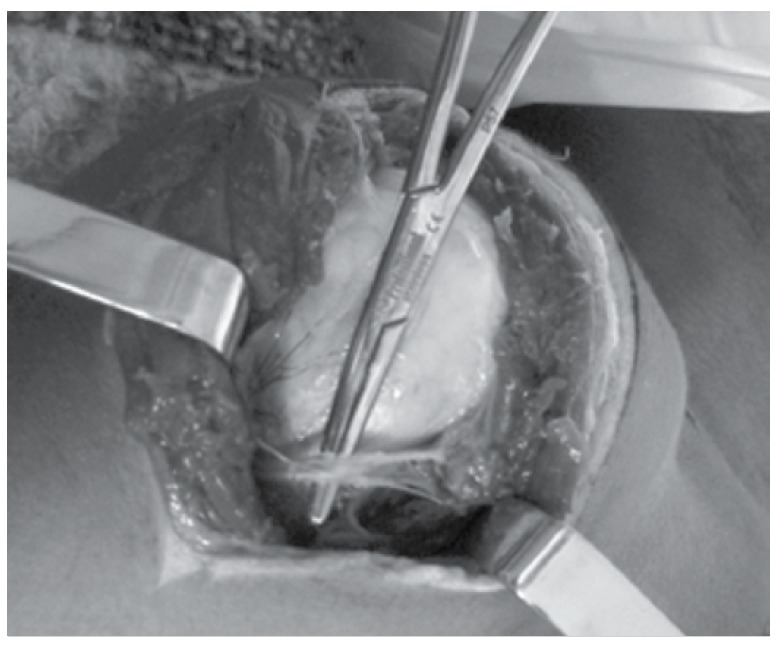
Dissection of the axillary nerve and its exposure (identified by the clamp).

Upon completion of the incision the anterior and middle portions of the deltoid muscle were identified. The dissection was made between these two portions to approximately five to seven centimeters from the anterolateral border of the acromion, with distal dissection of the axillary nerve. (Figure 2)

After identification of the axillary nerve, the deltoid was detached from the acromion to its anterolateral portion and the distance of that portion and the axillary nerve was measured with universal caliper (Digimess-São Paulo, SP). The length of the humerus was measured with a measuring tape from the top of the head to the lateral epicondyle in neutral rotation.

Statistical analysis was performed with SPSS software (Statistical Package for Social Sciences - Chicago, Il) version 13.0. The relationship between the measured distance of the axillary nerve and the length of the humerus and the relationship between this distance and body height was studied using the Spearman correlation analysis. The Anderson-Darling normality test was applied to verify whether the distance between the axillary nerve to the acromion had normal distribution and the Student t-test was applied to check for differences between the right and left shoulders.

This study was approved by the Research Ethics Committee of Faculdade de Medicina do ABC under registration 358/2006 issued on December 14, 2006.

## RESULTS

The right humerus length ranged from 28 to 33.5 cm (mean 30.30±1.53 cm) and the left from 27.5 to 33.5 cm (mean 30.45±1.67 cm). For both shoulders the distance between the axillary nerve and the anterolateral edge of the acromion ranged from 4.3 to 6.4 cm (average of 5.32±0.60 cm).

The Spearman correlation analysis showed that the distance of the axillary nerve increased with increasing body height (correlation coefficient +0.262) and increasing the length of the humerus (correlation coefficient +0.473), which was statistically significant only regarding the length of the humerus (p=0.026).

We observed that the posistive correlation between the distance of the axillary nerve and the humeral length was statistically significant (p<0.05). However, the correlation between the height of the specimens and the distance of the axillary nerve was not statistically significant (p=0.240).

**Figura 3.**
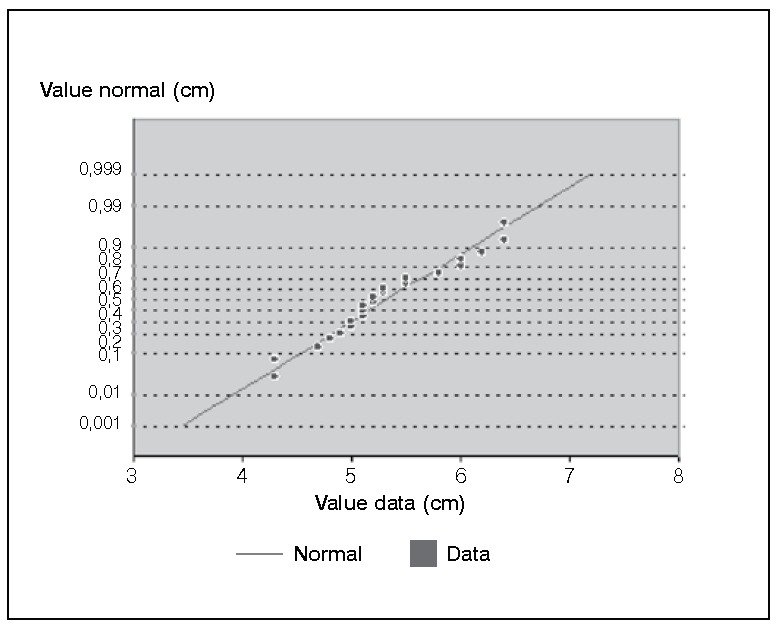
Normal distribution of distances between the axillary nerve and the anterolateral edge of the acromion on the right and left shoulders.

The Anderson-Darling normality test showed normal distribution of distances between the axillary nerve and the anterolateral edge of the acromion. (Figure 3)

There was no significant difference between the right and left shoulders using the Student t-test (p = 0.932).

## DISCUSSION

The injury of the axillary nerve is a possible and disastrous complication during shoulder surgery and results in severe impairment in shoulder function; thus, the study of its anatomical relationships is extremely important.^15^^,^ 16 There are several anatomical studies of the axillary nerve, especially about its correlation with other structures, which are necessary to prevent injuries.^6^^-^ 11 ^,^ 13 ^,^ 14 ^,^ 16 ^-^ 19

Bryan *et al.*6 measured the distance between the anterior branch of the axillary nerve and the distal limit of the anterior and anterolateral tract in 22 shoulders. These accesses were extended at least 5 cm beyond the edge of the acromion. The authors found that, on average, the distance between the axillary nerve and the distal limit of the previous operation was 0.65 cm and the distance between the nerve and the anterolateral access was 0.9 cm.

Ferreira Filho *et al.* 9 found that, on average, the distance of the axillary nerve to the lateral edge of the acromion was 7.18 cm on the right shoulder and 7.32 cm on the left.

Bono *et al.*,^18^ using posterior approach, studied the relationship between the axillary nerve and the humerus in 50 cadavers. The authors noticed that the distance between the nerve and the top of the humeral head was on average 6.09 cm. Furthermore, they found that the distance between the nerve and the surgical neck of the humerus was on average 1.72 cm.

Rocha *et al.* ^13^ dissected 20 shoulders from 10 cadavers to study the distance between the axillary nerve and the lower portion of the subscapular tendon insertion. Using deltopectoral approach, they observed a distance of 2.5 cm on the left shoulders and 2.6 cm in the right shoulders.

Cetik *et al.* 19 studied the distance between the axillary nerve and the anterior and posterior edges of the acromion, as well as the relationships between these distances and the length of the humerus. The authors dissected 24 shoulders and observed that the length of the humerus was on average 30.4±1.61 cm. The distance between the axillary nerve and the anterior border of the acromion was on average 6.08±0.45 cm and the distance of the nerve to the posterior border of the acromion was 4.87±0.26 cm on average. They concluded that above the axillary nerve there is a safety zone with variable distance form the lateral acromion edge according to the humeral length fo each individual. 

The number of patients undergoing reverse and resurfacing arthroplasty has grown steadly and many surgeons prefer the anterosuperior approach to perform these procedures.^1^^-^ 4 The relationships of the axillary nerve in the antero-superior approach are not documented in the literature and neither its distance from the anterolateral border of the acromion, which is exposed during approach. We decided to study the anterior approach in cadavers and analyze the distance of the axillary nerve to the anterolateral border of the acromion, as well as their relationship to the length of the humerus and body height in order to establish a safety zone for this approach.

We observed a normal distribution of these distances, on average 5.32±0.60 cm on both shoulders. Thus, there is a 95% confidence interval that the specimens had a distance to the axillary nerve that allows us to use the anterolateral edge as a reference frame. This study presents distances that differ from those found by other authors, probably because they used other different fixed milestones and studied different regions in the approach to the axillary nerve.

In addition, this study showed that there is a significant relationship between the distance of the nerve and the length of the humerus. We created a formula to estimate the distance of the axillary nerve to the acromion, based on the length of the humerus. The linear regression formula which correlates the axillary nerve to the length of the humerus is as follows:

Axillary nerve distance (cm) = [0.155 x humeral length (cm)] + 0.628

Using this formula, it was possible to estimate the distance and reduce the risk of axillary nerve injury in antero-superior approach.

## CONCLUSION

The distance between the axillary nerve and the anterolateral edge of the acromion is 5.32 ± 0.60 cm on both shoulders. This distance increases with increasing the length of the humerus.

This study shows that it is possible to know approximately where the axillary nerve crosses the anterior approach by the length of the humerus. In this way, it is possible to avoid complications through this approach.
